# The Application of Hough Transform and Canny Edge Detector Methods for the Visual Detection of Cumuliform Clouds

**DOI:** 10.3390/s21175821

**Published:** 2021-08-29

**Authors:** Aleksandr Lapušinskij, Ivan Suzdalev, Nikolaj Goranin, Justinas Janulevičius, Simona Ramanauskaitė, Gintautas Stankūnavičius

**Affiliations:** 1 Antanas Gustaitis Aviation Institute, Vilnius Gediminas Technical University, LT-08217 Vilnius, Lithuania; ivan.suzdalev@vilniustech.lt; 2 Faculty of Fundamental Sciences, Vilnius Gediminas Technical University, LT-10223 Vilnius, Lithuania; nikolaj.goranin@vilniustech.lt (N.G.); justinas.janulevicius@vilniustech.lt (J.J.); simona.ramanauskaite@vilniustech.lt (S.R.); 3 Faculty of Chemistry and Geosciences, Institute of Geosciences of Vilnius University, LT-01513 Vilnius, Lithuania; gintas.stankunavicius@gf.vu.lt

**Keywords:** thermals, cumuliform clouds, detection, UAV, soaring, Hough transform, Canny edge detection

## Abstract

The increase in flying time of unmanned aerial vehicles (UAV) is a relevant and difficult task for UAV designers. It is especially important in such tasks as monitoring, mapping, or signal retranslation. While the majority of research is concentrated on increasing the battery capacity, it is also important to utilize natural renewable energy sources, such as solar energy, thermals, etc. This article proposed a method for the automatic recognition of cumuliform clouds. Practical application of this method allows diverting of an unmanned aerial vehicle towards the identified cumuliform cloud and improving its probability of flying into a thermal flow, thus increasing the flight time of the UAV, as is performed by glider and paraglider pilots. The proposed method is based on the application of Hough transform and Canny edge detector methods, which have not been used for such a task before. For testing the proposed method a dataset of different clouds was generated and marked by experts. The achieved average accuracy of 87% on the unbalanced dataset demonstrates the practical applicability of the proposed method for detecting thermals related to cumuliform clouds. The article also provides the concept of VilniusTech developed UAV, implementing the proposed method.

## 1. Introduction

This century has shown a significant improvement in autonomous aircraft control systems, bringing forth numerous studies on the design and application of autonomous aircraft in various scientific fields [1]. The ability to fly without using its own energy resources is important for gliders, paragliders, and autonomous aircraft. Renewable energy resources, such as solar power and meteorological phenomena, such as thermals, permit to remain aloft longer, improving the efficiency as a result. The use of thermal flows with regard to autonomous aircraft flights is investigated already [2,3]. The main difficulty when forecasting thermals is the fact that they are invisible to the naked eye and other visual recognition equipment. Therefore, to forecast thermals, it is necessary to consider either the reason for their occurrence or the result of the occurrence of thermals. The reason for the meteorological condition is favorable for thermal flows and heat convection, as well as uneven heating of the Earth’s surface. Meanwhile, the result of the thermals is cumuliform convective clouds. Currently, autonomous aircraft flight in thermals occurs only incidentally as an aircraft inadvertently flies into a thermal flow [4]. The recognition and analysis of cumuliform clouds as they are growing is a reliable way to identify thermals.

Based on the location of clouds, it is possible to predict the places where a thermal is likely. Cumuliform clouds are formed by convection and rising air flows [5]. The active surface is heated by the sun. Hotter and lighter air volumes form over areas that are heated more. Affected by the principles of floatation, they move upwards. Gradually, separate volumes rise and converge into one, and a powerful rising airflow forms. Simultaneously, a myriad of smaller compensating downdrafts form in the periphery of the main flow. As a result, thermal convection occurs. The intensity of convection depends both on the non-uniformity of the surface and the degree of instability of atmospheric layers. Due to adiabatic cooling, convective (cumuliform) clouds form in the rising flows. The lower base of the clouds almost coincides with the level of condensation, whereas the top–with the level of convection [6]. Cumuliform clouds have the most active turbulence [7]. The turbulent zones in this environment can be identified visually [8]. We can presume that turbulence occurs in cumuliform clouds due to convection. As follows, cumuliform clouds, due to their visual shape, can be distinguished from other clouds in the sky by using image recognition methodologies.

The article aims to analyze the possibility of detecting cumuliform clouds and thermals below them by using methods of visual detection. The hypothesis is that it is possible to detect a growing cumuliform cloud by using image recognition algorithms without supervised cloud image-based learning. The proposed classifier could be used to divert an autonomous aircraft towards detected cumuliform clouds, thus increasing the possibility of flying into a thermal.

In Section 2, existing research papers were analyzed to get know what the solutions and results to the cloud classification problem are. While most existing cloud classifiers on ground-based camera images are based on supervised learning, the proposed approach will apply Hough transform and Canny edge detector methods. The models used are presented in Section 3, while the proposed method architecture is described in Section 4. The proposed solution does not require a cloud image dataset for model training while is able to achieve the same accuracy as existing supervised learning solutions. The results of the proposed method’s performance are presented in Section 5. As well, the paper presents an idea to use the proposed cumuliform cloud automated detection as a support system for UAV path planning in real-time, for flight time increase. The design of such a solution is presented in Section 6 and will be fully implemented in the future.

## 2. Related Work

The tasks of cloud recognition, classification, and tracking are important in meteorology, aviation, energetics, cartography, and reconnaissance [9]. Zhenzhou Peng suggests classifying clouds using images, obtained from an unmanned aerial vehicle (UAV), using thermals [10]. The UAV first must find and recognize the clouds in the sky, reject the images of objects on the ground, and classify the cloud as cumuliform.

Globally, several research studies, concerning cloud recognition and tracking, have been conducted. Cloud tracking technologies are important objectives in meteorology as well as for generating solar power. Currently, the two most popular methods for cloud tracking are used–the block comparison method and the variable optical flow method [10]. Satellite images or material from ground-based sky imaging devices can serve as a good source of cloud images [10]. When tracking clouds from several different points, it is also possible to determine their base above ground level [11].

Some studies [12] analyze automatic cloud classification based on images. The typical devices used for ground-based cloud classification are whole sky imager (WSI), total sky imager (TSI), and infrared cloud imager (ICI).

Local Binary Classifier (LBT) algorithms are employed for the classification. There are several subcategories of these algorithms, and each category has its advantages and disadvantages for image classification and recognition [12]. In ground-based cloud recognition analysis [13], authors use several different methods for cloud type classification: autocorrelation, co-occurrence matrix, edge frequencies, and length of primitives. The K-nearest neighbor and Artificial Neural Network algorithms were used for cloud type recognition too [13]. There are some research studies where image recognition algorithms are applied to satellite imaging to distinguish clouds from snowfields or mountain tops [10].

Long [14] analyzed automatic cloud recognition algorithms using images obtained from devices automatically photographing the sky. He suggested recognizing clouds based on the ratio of red and blue color in RGB channels of the pixels in the analyzed image. If this ratio is greater than 0.6, the pixel could be classified as one belonging to a cloud, otherwise, the pixel was attributed to the clear sky [14].

Another work [15] used optical as well as infrared ranges for cloud classification. The KNN classifier was employed for cloud classification. The accuracy of cloud classification in the work analyzed is at least 75 percent. The following cloud types were distinguished in the study: Cumulonimbus, Small cumulus, Clear sky, High cloud, Mixed clouds, Others, Overcast, Towering cumulus. The classified cloud image is transformed into a feature matrix using different transformations and filters: grey-level co-occurrence matrix (GLCM) statistics, Fourier Transform (FFT) power spectrum properties, and energy outputs from Laws and Gabor digital filter banks. The feature matrix is transferred to the KNN classifier.

The main papers on image-based cloud classification are summarized in Table 1. The summary illustrates different types of data sources used-satellite, ground-based total sky imager, infrared, digital camera images. At the same time, the used image dataset varies. There is no one standard, therefore even for the same data source, no unified, globally used dataset exists. This is partially related to the lack of a very wide dataset with separate portions of data for model training, validation, and testing. The absence of a publicly available ground-truth dataset complicates the comparison of different solutions. Therefore, most of the analyzed solutions rely on obtained result comparison with published results in other papers.

Other variations are the aim of the classification as well as the number of classes in the classifier. As there are no papers oriented to UAV flight time improvement, no analysis on specifically cumulative and non-cumulative cloud/sky identification was observed.

It is worth mentioning, the accuracy of ground-based camera cloud classification reaches up to 100% accuracy for some types of clouds, however, those solutions are based on supervised learning. Mostly k-nearest neighbor and convolutional neural networks are used for ground-based camera sky image classification. However, analyzing the average metrics (for all classes, not one only), the cloud classification accuracy from ground-based camera images mostly does not exceed 90%—the maximum values are mostly achieved by identifying clear sky only.

## 3. Research Methods

### 3.1. Canny Edge Detector Algorithm

The Canny algorithm can detect the edges of objects in images. This algorithm can also detect the edge of a cloud. The Canny operator was developed in 1986 by John F. Canny and uses a multi-stage wide spectrum algorithm for edge detection in images. John F. Canny studied and solved the mathematical problem of a filter, which is the most optimal based on the detection criteria, such as localization of minimums and obtaining several responses for a single edge. The algorithm consists of five stages: (1) blur; (2) finding the intensity gradients; (3) non-maximum suppression; (4) double threshold filtering; (5) hysteresis thresholding. The Canny detector reacts to the real edges of the image, and is resistant to false edges, by accurately detecting the edge curves of the elements, and also reacts to these curves only once, thus eliminating the impact of wide brightness change areas on the edges [25].

The Canny algorithm is susceptible to noise. One of the ways to diminish the effect of noise on the image is to use the Gaussian blur. This is a technique for image blurring, using a Gauss cell (3 × 3, 5 × 5, 7 × 7, etc.). The dimensions of the cell depend on the necessary level of image blurring. The smaller the dimensions of the cell, the less visible the effect of blur [26].

### 3.2. Hough Transform

Hough transform is a method for detecting lines and curves in a grayscale or color image. This method allows setting specific parameters for the subgroups of curves and ensures the detection of the pre-set subgroup of curves in the image. Various types of curves can be detected, such as straight lines, arcs, circles. It also allows for detection based on a pre-set template. The Hough transform algorithm uses an accumulator space, the number of dimensions of which corresponds to the number of unknown parameters in the equation to detect the curve subset. For example, when detecting the curve y = m*x + b, the values for the parameters of m and b for each line must be found. In this case, the values are accumulated in the element array A [M, B], and show the probability of the existence of lines corresponding to the equation y = m*x + b in the analyzed image, where M and B are the discrete values of m and b. The Hough transform is used in the areas of meteorology and hydrology [27,28]. However, it has not been used in the context of detecting cumuliform clouds.

### 3.3. The Kalman Filter

The Kalman filter estimates a joint probability distribution over the variables of an object under analysis, using a series of measurements observed over time. This allows minimizing the impact of noise on the parameter under analysis and obtaining smaller errors of measurement of the parameter in the past, present, and future. The Kalman filter is a recursive filter that, according to the obtained measurements, carries out an optimal assessment of a momentary state of a linear dynamic system, which is affected by Gaussian noise with a normal distribution. The Kalman filter is mainly used for the assessment of the process state x ∊ R, where the process is expressed in linear differential equations (vector and matrix forms) [29].

### 3.4. Detection of Clouds and Ground Surface Based on the Color Balance

By employing an analysis of the ratio between the red and blue channels, it is possible to make a distinction between pixels that belong to the clouds or to the sky itself [14] According to Long et al. [14], not only do clouds have their distinct ratio of color balance. A comparison of the average values of the colors allows distinguishing clouds from the surface of the Earth [14]. Zafarifar and Weda study [30] also note that the horizon can be detected by analyzing the colors of the image pixels. Complex horizon detection algorithms, analyzed in the study by [31], could be used. However, it is stated that these algorithms require from 0.3 to 61.1 s for operating on 1024 × 768 resolution pixel images. This horizon detection speed is very slow, especially, since it is not necessary to detect the horizon very accurately when the computation resources can be used better for the detection of cumuliform clouds. A premise can be set forth that the comparison of other color ratios allows detecting the horizon faster than within 0.3 s or eliminating the horizon by simply disregarding the lower part of the image.

## 4. Description of the Suggested Hough Transform and Canny Edge Detector Based Cumuliform Cloud Detection Method

**Figure 1:**
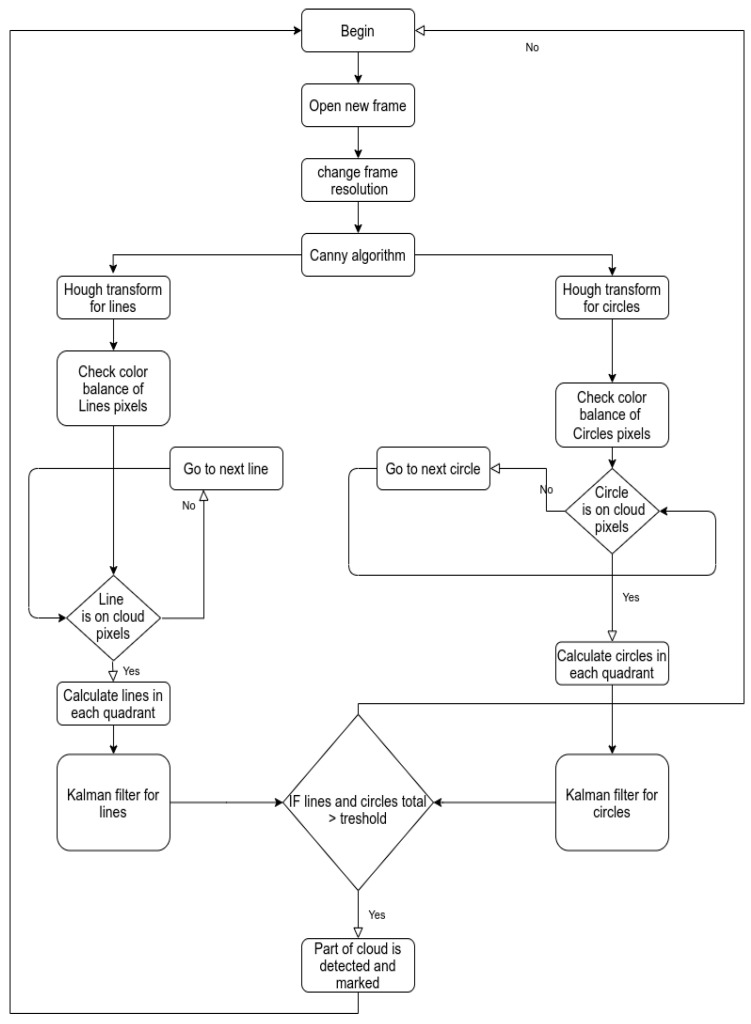
Diagram of the proposed algorithm.

As previously mentioned, due to the shape of cumuliform clouds affected by convection, in theory, the use of the Hough transform would allow identifying the places with cumuliform clouds in the images. To employ the Hough transform, the image has to be manipulated in a way that only the edges of the objects are left. The Canny algorithm was used for this purpose. The OpenCV package [32] was used in the experiment. In essence, the cloud detection algorithm consists of the processing of the image using the Canny algorithm, followed by the application of the Hough transform for calculating the separately detected lines and circles. During the experiment, the frame was converted into a 4 × 4 square matrix, with a total of 16 quadrants. The computation of lines and squares is not carried out for the full frame, but separately for each quadrant of the frame. Since the number of lines and circles is different in different quadrants, the Kalman filter was applied individually to each quadrant, by smoothing the number of detected lines and circles. In the final stage, a threshold function was applied, to determine whether the number of detected lines and circles in a quadrant exceeds a certain limit. If the threshold function returns a positive result, the quadrant is attributed as belonging to a cumuliform cloud. It is also possible to determine how close a cloud is. If the camera is lower than the cloud base, closer clouds will be found at the top of the image, whereas clouds further away will be found in the lower part of the image. The top quadrants, in this case, will have closer clouds, and the lower quadrants next to the horizon will hold clouds further away. This way, the UAV may choose closer prospective cumuliform clouds to use the thermals below them for autonomous soaring. Using these premises, an algorithm was formulated, the aim of which is to detect cumuliform clouds against a background of the sky or other clouds. The diagram of the algorithm is illustrated in Figure 1.

All images used in the model, irrespective of the source of the image, must be made similar in resolution since the dimensions of the detected objects must fit into certain pre-set boundaries. The resolution of the images was set to 1024 pixels horizontally. The resolution in pixels vertically was calculated according to Equation (1).

(1) $$\mathrm{NewHeight}=\frac{\mathrm{OldHeight}\cdot \mathrm{NewWidth}}{\mathrm{OldWidht}}=\frac{\mathrm{OldHeight}\cdot 1024\mathrm{px}}{\mathrm{OldWidht}}$$

The second stage is the application of the Canny algorithm to detect the edges of the image. The quality of the operation of the whole algorithm depends on the quality of this stage. Only the edges of the clouds necessary for this step are identified in this stage, rejecting noise and other small details present in the image. With the application of the Canny algorithm, the image of the cloud will appear as a combination of curves and straight lines against a black background. This generated image can be used for detection by applying the Hough transform to search for curves and circles in the image. The parameters of the Canny algorithm, used for the experiment, are listed in Table 2. These are the standard parameters of the Canny algorithm while values were selected experimentally, taking into account the collected dataset and using its statistical data [33]. This is not an optimal method to estimate the lower and upper thresholds, but it is enough for basic functionality, while will be optimized in the future.

The minimum and maximum dimensions of the detected primitives are set in the Hough transform algorithm. If, for example, the resolution in one instance is 400 × 400 pixels, whereas in another instance 4000 × 4000, the difference between the dimensions of the objects suitable for detection will be 10 times greater. Therefore, a possibility for a false positive or a false negative result of an object detection occurs.

The circle and line shapes have different characteristics, therefore suggested Hough transform algorithm parameters were set to circle detector (see Table 3) and line detector (see Table 4). The following parameters provide the dimensions of the detected objects. It was selected taking into account the size of the image and possible distance from the camera to the taken clouds (for example the variation of cloud size varies in the image, based on image size, therefore the circle radius ranges can be set based on it). To adjust the parameters, grid-based parameter optimization could be executed, however, it is time and resource-consuming. Therefore, in this research intuitive parameter values were selected.

**Figure 2:**
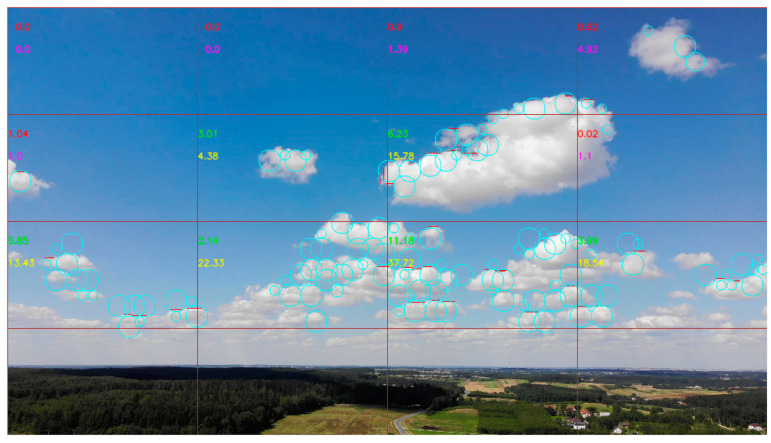
Visual illustration of the output of the proposed algorithm, where image segmented to 16 elements and the bottom four elements are eliminated from the analysis.

It is not necessary to detect objects below the edge of the horizon. As a result, the edge of the horizon was eliminated by separating the image into 16 quadrants, with the bottom four quadrants, where the horizon lies, not analyzed by the algorithm (Figure 2).

The next stage is eliminating the lines and circles that do not belong to the pixels of a cloud. Cloud pixels are identified using the method previously described in [14]. The cloud detection algorithm operates using the following method. First, the primitives (lines and circles) are detected using the Hough transform. The primitives have their central coordinates, x, y accordingly. A pixel within the coordinates of the detected primitive (line or circle) is chosen, and the three color channels of this pixel are extracted—red (r), green (g) and blue (b). Each channel is coded by a number from 0 to 255, where 0 represents the lack of color, whereas 255 represents the greatest possible value of the color. Then, it is assessed whether all three channels have values that are greater than 120—which stands for the empirically derived threshold. This threshold ensures that the algorithm discards dark pixels. Then, based on the research by Long [14], the ratio between the values of the red and blue channels is assessed, comparing it to a 0.6 threshold. The pixel is attributed to a cloud if all four criteria are met: red channel value is greater than 120, green channel value is greater than 120, blue channel value is greater than 120, and the ratio between the red and blue channel values is greater than 0.6. Otherwise, the pixel is attributed to a clear sky.

The cloud position has to be identified in a certain area identified by coordinates. Image fragmentation into numbered quadrants was used in the experiment, and the detected cloud position was attributed to the quadrants. Since the algorithm can be used for video feed as well as images, the number of primitives in each frame, detected using the Hough transform, will differ. To smooth the number of primitives detected in each quadrant, smoothing filters are used for each quadrant separately. The Kalman filter is applied for this purpose to each quadrant separately to separately smoothing the number of lines and circles.

After the straight lines and circles in the quadrant are detected and calculated, it must be determined whether there is an image of a cumuliform cloud in this quadrant or not. This is carried out by using a threshold function. The sum of all the lines and circles in a quadrant is fed into the function. If the sum is higher than the preset 7-unit value, the algorithm identifies the quadrant as containing a cumuliform cloud.

Figure 2 illustrates the image, divided into 16 quadrants. The lowest four quadrants are not analyzed, as they contain the horizon line. Meanwhile, the other twelve quadrants are analyzed to identify circles and straight lines in them. Identified circles (presented in light blue color) and straight lines (presented in red color) are presented just for visualization. The classification result is executed based on the number of these primitives in each quadrant. Therefore, for visualization, each analyzed quadrant has two decimal numbers, presented in the left top corner of each analyzed quadrant. The upper number represents the number of lines, while the lower one–number of circles. The numbers are not integers, as the whole image is analyzed and the quadrant might contain just some portion of identified primitive, not the whole one. If the quadrant is classified as containing cumuliform clouds–these two numbers are presented in green and yellow. In other cases–the numbers are presented in red and pink. Therefore, in the example of Figure 2, only six quadrants are classified as containing cumuliform clouds, six quadrants are classified as not containing cumuliform clouds (clear sky or contain other types of clouds).

It must be noted that the 7-unit threshold is an empirical value, that must be set based on the following:

1. Parameters of the Canny algorithm.
2. Parameters of the Hough transform for lines detector.
3. Parameters of the Hough transform for circles detector.

It is necessary to adjust the cloud detection threshold for the different parameter settings mentioned above. It is also likely that the threshold has to be adjusted concerning the time of day, meteorological conditions, and the characteristics of the camera.

## 5. Results and Discussion

### 5.1. Description of the Dataset

To approve the suggested method, an experiment for the automatic detection of cumuliform clouds was set up. A dataset of 6456 images of different types of clouds was collected. The images were generated from a video feed provided by a drone. When collecting the data, the drone was flown at 120 m, under different meteorological conditions, and at different times of day, to collect a varied array of cloud images. During data collection, the drone performed a 360-degree rotation along the vertical axis, thus scanning the sky around itself. The same drone was used for the collection of images in the dataset without changing the main parameters of the camera, or the resolution. However, the videos were made in three different locations during a period of 15 days. The dataset was composed of situations, when autonomous soaring is possible (the worm period on daylight), as the method is dedicated to detecting cumuliform clouds during gliding.

The data from the drone were converted into 1024 × 576 px size images, which were then segmented into 16 images of 256 × 144 px (four lines and four columns). The detection of cumuliform clouds was carried out simultaneously with the segmentation of the image by using the described computer vision method. As a result, the images for the dataset were obtained together with the numbers of circles and lines, detected by the cloud detection algorithm. Thus, the detection of contrasting lines and circles was carried out in real-time together with the collection of the images for experimental analysis. The lower line consisting of four images was eliminated from the dataset, as most of it was attributed to the horizon (Figure 2).

To approve the accuracy of the algorithm, the images were classified into cumuliform and other types of clouds using expert analysis. Experts were trained by using collected training material. The most prominent features of cumuliform clouds, as well as the differences from other types of clouds, were described in the training material. Examples of the training material, the developed code, and the dataset can be found at github: https://github.com/ivansuzdalev/clouds_reco_canny_hough (accessed on 25 August 2021). After the experts were acquainted with the training material, the data were classified. The classification was carried out by three trained experts. A logic “1” was attributed to the images with a cumuliform cloud or its part. Values of images classified by experts:

- Expert no1: classified 1737 as a cumuliform cloud image of 6457 images.
- Expert no2: classified 1851 as a cumuliform cloud image of 6457 images.
- Expert no3: classified 1725 as a cumuliform cloud image of 6457 images.

The results of the three experts were averaged. In the next step, the collected visual data were fed into the cloud detection algorithm. As mentioned previously, the images of cumuliform clouds are characterized by contrastive curves. Therefore, an image within which a certain threshold number of detected primitives (lines and circles) is detected may be classified as an image of a cumuliform cloud. The threshold number of simple primitives is set using a threshold function. In the experiment, the threshold of seven detected primitives (circles and/or lines) was set for the classification of an image as containing a cumuliform cloud. The results of the algorithm were collected into a database side by side with the fragments of the images for experimental analysis.

### 5.2. Assessment of Cumuliform Clouds Detection Results

The initial dataset was split into several subsets to assess the accuracy of the algorithm. Such a splitting allows method stability estimation as accuracy metrics can be analyzed in different sets of images. This illustrates more realistic, rather than synthetic conditions (when the number of each class is the same). The splitting of the initial dataset gave us the possibility to calculate the accuracy error rate. The splitting of the dataset into smaller subsets, provided in Table 5.

The following metrics were calculated:

- True positive (TP)—the number of cumuliform cloud images accurately detected by the algorithm.
- True negative (TN)—the number of images without cumuliform clouds detected accurately by the algorithm.
- False positive (FP)—the number of images that the algorithm inaccurately identified as cumuliform cloud images.
- False negative (FN)—the number of images that the algorithm inaccurately identified as not being cumuliform cloud images.

The results of this stage are provided in Table 6.

Based on the confusion matrix, the following metrics of the operation of the algorithm were calculated:

- Sensitivity or true positive rate (TPR) measures the proportion between correctly identified cumuliform cloud images (TP) and all cumuliform cloud images (P), which are equal to the sum of true positive and false negative (2).

(2) $$\mathrm{TPR}=\frac{\mathrm{TP}}{P}=\frac{\mathrm{TP}}{\mathrm{TP}+\mathrm{FN}}$$

- Specificity or true negative rate (TNR) measures the proportion between correctly identified non-cumuliform cloud images (TN) and all non-cumuliform cloud images (N), which are equal to the sum of true negative and false positive [34] (3).

(3) $$\mathrm{TNR}=\frac{\mathrm{TN}}{N}=\frac{\mathrm{TN}}{\mathrm{TN}+\mathrm{FP}}$$

- The positive predictive value (PPV) (4) and negative predictive value (NPV) (5) present the proportion between correctly identified cumuliform (positive, TP)/non-cumuliform (negative, TN) cloud images and the total number of positive/negative records [35].

(4) $$\mathrm{PPV}=\frac{\mathrm{TP}}{\mathrm{TP}+\mathrm{FP}}$$

(5) $$\mathrm{NPV}=\frac{\mathrm{TN}}{\mathrm{TN}+\mathrm{FN}}$$

- Accuracy (ACC) denotes the proportion between correctly classified records (TP and TN) and all cloud mages (6). Therefore, high accuracy is associated with both high precision and high trueness [36].

(6) $$\mathrm{ACC}=\frac{\mathrm{TP}+\mathrm{TN}}{P+N}=\frac{\mathrm{TP}+\mathrm{TN}}{\mathrm{TP}+\mathrm{TN}+\mathrm{FP}+\mathrm{FN}}$$

- Balanced accuracy (BA) is basically an average between true positive rate (TPR) and true negative rate (TNR) (7). It is used to evaluate how good a binary classifier is. Its biggest usage area is with imbalanced classes when one class has a bigger number of records in comparison to the second one [37].

(7) $$\mathrm{BA}=\frac{\mathrm{TPR}\;+\;\mathrm{TNR}}{2}$$

- F1 Score (F1) in binary classification measures the test’s accuracy. It is calculated as a double proportion between the production of positive predicted value (PPV) and true positive rate (TPR) and the sum of those two [38] (8).

(8) $$F1=2\times \frac{\mathrm{PPV}\;\times \;\mathrm{TPR}}{\mathrm{PPV}\;+\;\mathrm{TPR}}=\frac{2\mathrm{TP}}{2\mathrm{TP}\;+\;\mathrm{FP}\;+\;\mathrm{FN}}$$

All these metrics were used to reflect different parameters of the obtained results. The results of all these metrics are presented in Table 7.

### 5.3. Discussion on Cumulus Cloud Detection in Dedicated Dataset

In the analyzed datasets, the worst accuracy (ACC) result of the suggested algorithm was 0.75 or 75 percent. In one of the samples, the accuracy reached 0.97 or 97 percent. In the whole data sample, the accuracy reached 0.87 or 87 percent. The fluctuation of the accuracy across samples shows that the dataset was not well balanced. This conclusion is also supported by the Balanced Accuracy data. In the data samples from 1000 to 2500 and 2000 to 3500, the BA reaches 0.6 and 0.74. These data samples contained images of clouds that were not likely to generate thermals. Thus, it can be stated that the algorithm has greater accuracy when the cloud images have clear edges.

TPR values variance also suggest that the dataset was not well balanced. In the subset with clouds not suitable to generate thermals, the TPR is only 32 percent, whereas in the subsets with cumuliform clouds, the TPR was higher than 80 percent. This is supported by the PPV data, in the sample from 1000 to 2500 images, the PPV only reaches 0.16 or 16 percent.

Meanwhile, TNR variations are not as high and gain 0.90 value for the whole dataset. The TNR values for different subsets of the dataset vary from 78 to 99%. This shows the proposed method is suitable to eliminate non-cumuliform clouds.

Taking into account the TPR, PPV, F1-Score values drop drastically for some subsets, while TNR and NPV values have smaller variations it could be said the achieved classification accuracy can be increased by adjusting the detected primitive’s threshold for a specific dataset. However, the lowest achieved accuracy of 75 percent obtained in the experiment is similar to the accuracy of the algorithm provided in the study by Emal Rumi [15], Min Xia et al. [19], Lyan Ye [21], Yang Xiao et al. [22], and other similar works. This illustrates non-cloud image supervised learning solutions can achieve analog results as those, trained with labeled cloud image datasets. As well, the obtained overall 87% accuracy demonstrates the practical applicability of the proposed method. It could be even increased if additional dataset and method tuning would be applied.

Talking about the limitations of the method–the method was tested with the dataset, representing different situations, oriented to conditions, suitable for UAV soaring and thermal existence. It was carried out intentionally, as the application of the method is planned for thermal search. Therefore, no photos were included to present sky images at night, in winter, or in other nonstandard conditions. For such nonstandard conditions, the method parameters should be adjusted to take into account the image color palettes at night or other specific requirements. Additionally, the camera location on the UAV has to be placed stable and pointing straight. This is important as the method at its current state searches for the horizon line on the lower quarter of the photo. If the horizon will be above the ¼ line, it will be analyzed as the sky, therefore some buildings or other objects can be treated as cumuliform clouds. In cases, where camera location is strictly pointed down, the proposed method parameters should be adjusted to define the possible ranges of the horizon.

Summarizing–the method uses some parameters, which can be adjusted for better performance. As the method is not based on supervised learning, the parameters should be estimated experimentally or by using some optimization methods. Using the same parameter values in different situations, the proposed method may not demonstrate suitable results in other situations. Therefore, before using the method for cumuliform cloud classification with images of different cloud/sky conditions, some sample images should be used for testing–to estimate the parameter suitability. This might require some adjustment for nonstandard conditions, but at the same time, it adds method adaptability, when adjusting the parameters, the method can be adapted for some very specific situations.

It should also be taken into account that the method is dedicated to the classification of cumuliform clouds from other clouds or clear sky for UAV flight path planning. It may be difficult to generalize it easily in other scenarios such as different cloud type classification, or in conditions unsuitable for UAV flight (night, low altitude flights, etc.).

### 5.4. Method Testing with Other Cloud Datasets

The existing and publicly available cloud datasets are poorly labeled for cumuliform clouds (it is related to different cloud classification categories as well as dataset purposes). Therefore, it is difficult to evaluate the proposed method on other existing datasets. One of the most used ground-based camera cloud image dataset is SWIMCAT [39]. It has six cloud classes: Clear sky; Patterned clouds; Thin white clouds; Thick white clouds; Thick dark clouds; Veil clouds. Clear sky and veil clouds have a clear difference from cumuliform clouds. These two classes can be assumed as non-cumuliform cloud/sky examples. The other four classes have both cumuliform and non-cumuliform cloud samples. Therefore, it requires relabeling to present cumuliform and non-cumuliform cloud samples.

By applying the proposed method, the extended SWIMCAT cloud dataset images were processed and classified with the proposed method to cumuliform and non-cumuliform (see Table 8). This comparison does not allow method accuracy estimation but provides some guidelines on whether the method is sensitive to sky geographical area and photo source.

The results illustrate that all the images from the two clearly non-cumuliform cloud classes (clear sky and veil clouds) are classified as non-cumuliform. This proves that the proposed method is 100% accurate in eliminating clear sky and veil clouds from cumuliform clouds. Meanwhile, the results of the other four classes are hard to evaluate, as those SWIMCAT dataset classes contain both cumuliform as well as non-cumuliform cloud samples.

## 6. Application Concept and Practical Implementation

The proposed cumulative cloud detection method achieved good accuracy for visual sky classification to suitable areas for UAV path. Therefore this section describes the concept and idea of practical implementation in UAV. The UAV and integration principles of the proposed cumulative cloud recognition method are provided. Meanwhile, real path planning solution implementation and experiments for flight time increase are planned in near future.

### 6.1. UAV Preparation for Usage of the Proposed Cumulative Cloud Detection

The cloud detection flights should be performed following the suitable for them synoptic situations: no frontal cloud systems-only thermal convection available, warm half of the year and midday and/or afternoon hours; the predictions of widely used instability indices (threshold values) for the flight sites should be used: Convective available potential energy (CAPE), 10–500 J/kg and K index, 15–25 K. There are cases where the thermal activity is high, but there are no cumuliform clouds. In this case, predicting thermal flows based on visual data is impossible, and in such cases, the use of this algorithm is pointless.

**Figure 3:**
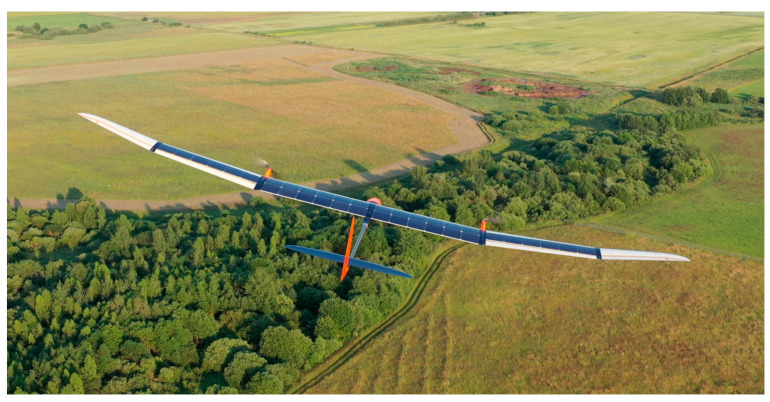
UAV, designed in VilniusTech university for autonomous soaring.

A UAV designed by VilniusTech and powered by renewable energy will be used for concept implementation. This UAV can stay aloft for a long time and can use thermals due to its high aerodynamic efficiency (Figure 3).

The specifications of the UAV which will be used for the experiment are presented in Table 9. However, the search for thermals is not automated yet, therefore the proposed method takes place and will be integrated in the future.

**Figure 4:**
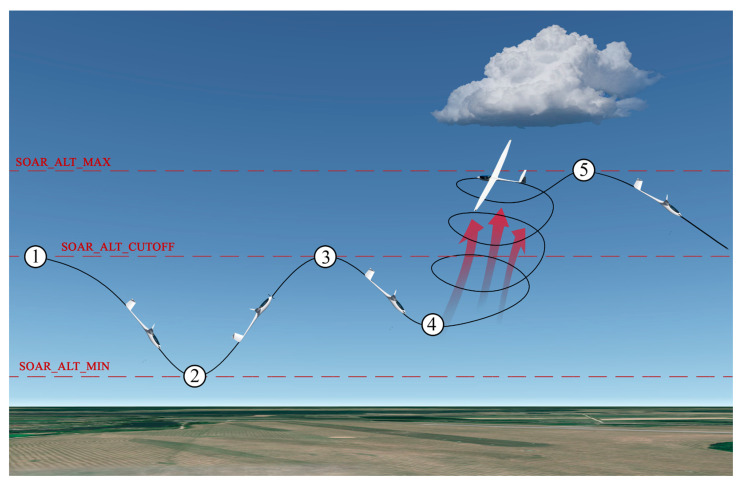
Stages of Ardu Soar algorithm for flights in thermals.

The UAV uses the open-source ArduSoar algorithm for thermal centering to climb when accidentally flying into a thermal [4]. All stages of ArduSoar algorithm are provided in Figure 4. UAV starts autonomous soaring during an autonomous mission then the ArduSoar algorithm is enabled (stage 1). UAV turns off his engine and starts gliding. If during descent, no thermal is detected and the lowest altitude SOAR_ALT_MIN is reached, UAV turns on its engine and starts climbing until it reaches SOAR_ALT_CUTOFF altitude (stage 2), and starts gliding again (stage 3). If during gliding, the thermal is detected, UAV triggers loiter mode to center the thermal and climb in it with no engine (stage 4). If SOAR_ALT_MAX altitude is reached, UAV stops loitering and starts to glide to the next waypoint. To improve the probability of flying into a thermal, the course of the UAV towards a prospective, the growing cumuliform cloud can be changed by the cloud detection algorithm during stages 1–4. The same technique to divert gliders to the nearest cloud is also used by glider and paraglider pilots for remaining aloft longer. Additionally, during these stages, any useful missions, such as surface monitoring can be performed. For the steady flight at the same altitude for such missions as mapping, autonomous soaring algorithms can be paused at any time. Virtual geo-fences can be used to keep the aircraft in the desired area and prevent it from flying out. Meanwhile, for tasks to get from point A to point B, the idea of path planning should be modified to take into account both the target as well as possible paths to it.

**Figure 5:**
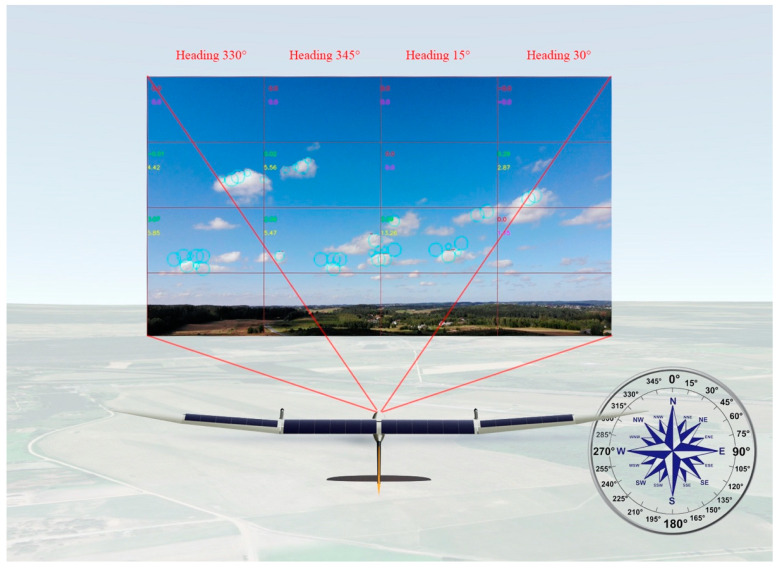
Practical implementation of the concept for cloud detection and diverting a UAV.

To benefit from the proposed cumuliform cloud classification solution, a camera, which collects the images ahead of the UAV in real-time, is to be mounted on the front of the UAV. Image processing according to the proposed method is to take place in a companion computer connected to the autopilot and magnetometer. Knowing the UAV heading from the magnetometer data as well as the angle of the camera, it is possible to attribute a specific heading for each image column that would lead towards a cloud. Figure 5. illustrates the prototype implementation of the concept for cloud detection and diverting of the UAV towards it. The image from the UAV covers a 60-degree area in front of it. Therefore, a wider range camera, multiple cameras, or UAV direction correction is needed to cover a wider area of cumulative cloud search. Additionally, Figure 5 illustrates the camera orientation met with proposed method settings, where the lowest quarter of the photo presents the horizontal line, therefore can be eliminated from the search.

The heading to the cloud can be prioritized based on the number of simple primitives found in a certain quadrant. Lower images closer to the horizon will have a lower priority since the clouds detected in them will be further away from the UAV, a higher priority will be attributed to the clouds closer to the UAV. Once the cloud was detected and a heading towards it was determined, the companion computer will send the autopilot a command to continue on this heading until the UAV flies into a thermal.

### 6.2. Simulation of UAV Flight Time

The applied ArduSoar thermalling controller for resource-constrained autopilots [4] is able to take advantage of thermals. If the UAV detects a thermal while gliding down, it takes advantage of it and goes into the loitering stage. During this stage, it continuously estimates the thermal center and follows it by spinning circles around it. Loitering increases the altitude of the UAV. The loitering stage is switched off to the gliding down stage when the maximum altitude is reached (SOAR_ALT_MAX) or the thermal effect is not detected anymore. This helps the UAV to gain altitude and increase the potential energy to glide down further.

A pilot with remote control and thermal identification skills executes the search and UAV pointing to possible thermals. Meanwhile, automated solutions rely on random hits of thermals in their gliding path. To identify the possible effect of visual cloud detection on UAV flight time, multiple simulations were executed.

To simplify the task, UAV missions with the engine off are analyzed only (gliding down or loitering stages only). The task for the UAV is simplified to fly from point S towards point F as long as it is able. The distance between S and F is 1000 m. The initial data for the simulations generated based on multiple experiments (log of example experiment are provided in https://ayvri.com/scene/ykxw3l4759/ckmdbe8rl00043a6h7u847ynk (accessed on 25 August 2021)), with Pixhawk autopilot and ArduSoar code equipped UAV:

- The cutoff altitude (SOAR_ALT_CUTOFF) of the UAV is 185 m.
- The maximum reached altitude (SOAR_ALT_MAX) is set at 1000 m.
- The minimum altitude before the engine will be turned off (SOAR_ALT_MIN) is set to 100 m.
- The average UAV speed is 13.6 m per second (standard deviation 4.204).
- The average altitude reduction while gliding down without thermal is 0.367 m per second (standard deviation 0.0048).
- The average altitude gain (climb rate) while loitering in type 1 (not powerful, reaching 300 m high only) thermal is 0.293 m per second (standard deviation 0.0102).
- The average altitude gain while loitering in type 2 (powerful, reaching 800 m and higher) thermal is 0.739 m per second (standard deviation 0.0172).

For simulation purposes, the thermal center is fixed (wind influence is eliminated as the wind can be of different directions as well as speed). Camera analysis is a 30-degree angle to both sides of its view. Both flight time and distance to the finish point are estimated.

**Figure 6:**
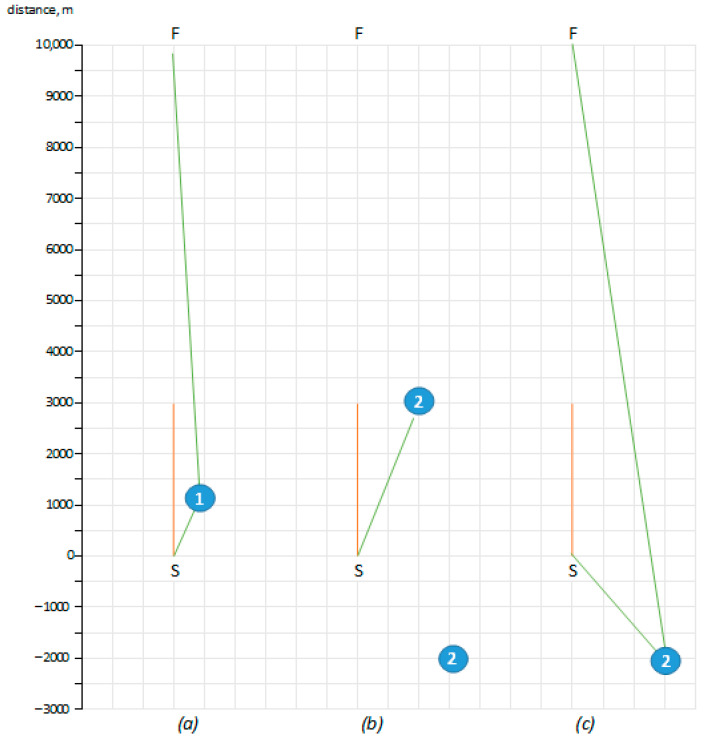
Example situations for simulation, where (**a**) illustrates type 1 thermal within 1320 m from the start in the range of UAV view angle; (**b**) illustrates type 2 thermal within 3200 m from the start in the range of UAV view angle and type thermal out of UAV view range; (**c**) illustrates type 2 thermal in the opposite direction of the finish when UAV analyses 360-degree view.

The comparison of cumuliform cloud detection-based thermal search (green lines) and direct flight with a hope to hit thermals (red line) is presented in different situations. The situations were generated to present a variety of possible outcomes (see Figure 6). Example (a) illustrates a very basic situation, when type 1 thermal is in the UAV view range and within UAV possible gliding distance (1320 m in ~25-degree angle). The thermal allows the UAV to increase the altitude to 300 m from 150, which is almost enough to reach the finish point. The total UAV flight time increases from 232 s to 1156 s, where 514 s were dedicated to gain altitude in the thermal (see Table 10).

Situation (b) illustrates the direction to possible thermal, without taking into account the distance to the thermal. It might increase the distance to the finish–the UAV floats to the right of the straight line to the F but is not able to reach the thermal and finishes its flight without reaching the F. It also illustrates that the UAV should view the whole 360 degrees to find other possible thermals closer to it, not to be limited to the 60 degrees only, as shown in Figure 5. Therefore, situation (c) illustrates the first 2.5 s are dedicated to overview the sky and choose the closest thermal in opposite direction to the F. As it is a type 2 thermal, the UAV gains 800 m altitude, which is enough to reach the finish and even go back to the S. The need to rotate for an overview of the whole sky is not needed, as the UAV flies in circles during the loitering stage, therefore the whole sky view can be constructed at the time.

The several simulated situations reveal the identification of possible thermal within the reachable distance might drastically increase the flight time and flight distance. At the same time, it revealed the need for a 360-degree view for the identification of suitable thermals. Taking into account the real-world situation is more complex because of cloud and thermal movement, the cumulus cloud detection should be implemented to work in real-time. This would present the current situation, as well the movement could be taken into account for distance to cloud calculation.

As well it is worth mentioning the thermal search-oriented solution is not suitable for UAV tasks, where some objects must be tracked–the direction to detected thermal might eliminate the detected object out of wight. Therefore, it is more suitable for non-time constrained tasks, where some points must be reached or the area within a given territory must be overviewed.

## 7. Conclusions

The performed literature analysis has revealed the possible applicability of Hough transform and Canny edge detector methods for detecting cumuliform cloud, which is in many cases related to thermals, that can be used for extending the flying range of UAVs if a detection mechanism is deployed onboard. It was also noted that the noted methods have not been used for the task before and other methods currently used for cumuliform cloud detection do not guarantee a high detection rate or require rather high calculation time.

The dataset, composed of 6457 images, containing and not containing cumuliform clouds collected by UAV, was created and data pre-processing was performed. All the images in the dataset were marked by three independent experts.

The Hough transform and Canny edge detector-based cumuliform cloud detection methods were proposed, as well as threshold parameters, which were defined to automatically decide on the presence or absence of cumuliform clouds in an image. The method was used against the prepared dataset and the method results were compared with experts’ evaluation. The average detection accuracy has reached 87%, which is rather promising, especially taking into consideration that the dataset is not yet well balanced, and further improvement of the dataset and method parameters could increase the accuracy. Still, even current results show an accuracy increase compared to the previously used methods.

The concept of the practical method implementation on the VilniusTech developed UAV is also briefly described, which includes the use of the front camera for making sky pictures, companion computer for cloud recognition, and its connection to the autopilot and magnetometer, thus directing the UAV to the most possible thermal positions.

## Figures and Tables

**Table 1:** Summary of image-based cloud classification solutions and their results.

Authors	Aim	Data Source	Used Method	Dataset	Accuracy
Z. Peng et al. [10]	Cloud movement identification	ground based TSI	Support Vector Machine for cloud area identification	Custom dataset, 8 TSI images for validation	Mean absolute error (MAE): 3.3–22.5
S. Liu and Z. Zhang [12]	Classification of 7 sky/cloud types	ground based TSI	Wireless sensor network	Kiel and IapCAS-E datasets	Accuracy (ACC) with different datasets: 83.21 and 78.94
Z. Zhang [16]	Classification of 7 sky/cloud types	ground based TSI	Convolutional neural network (CNN)	MOC_e, IAP_e, and CAMS_e datasets	Accuracy (ACC) with different methods: from 72% to 79%
S. Liu [17]	Classification of 7 sky/cloud types	ground based TSI	Convolutional neural network (CNN)	Custom dataset with 3711 records	Accuracy (ACC) for different methods: from 75% to 93%
J. Drönner et al. [18]	Classification of 5 cloud/land classes	Satellite images	Convolutional neural network (CNN)	Visible and Infrared Imager (SEVIRI) on satellites	Accuracy (ACC) for different cases: from 89% to 94%
E. Rumi et al. [15]	Classification of 8 sky/cloud types	ground-based camera and infrared camera images	k-nearest neighbor classifier	Custom dataset of 13,197 records	Accuracy (ACC) for a different month: from 67% to 90%
M. Singh and M. Glennen [13]	Classification of 5 cloud types	ground-based camera images	k-nearest neighbor and neural network classifiers	Custom dataset of 3167 images	Accuracy (ACC) with different folds: from 20% to 70%
M. Xia et al. [19]	Classification of 4 sky/cloud types	ground-based camera	k-nearest neighbor and extreme learning machine classifiers	Custom dataset with 840 records	Accuracy (ACC) for different sky types: from 77% to 100%
Y. Wang et al. [20]	Classification of 5 sky/cloud types	ground-based camera	Different neural networks	SWIMCAT dataset	Accuracy (ACC) for different methods: 71% and 85%
L. Ye et al. [21]	Classification of 6/9 sky/cloud types	ground-based camera	Convolutional neural network (CNN)	ImageNet dataset with 1000 records	Accuracy (ACC) for different methods: 72% and 98%
Y. Xiao, et al. [22]	Classification of 6/9 sky/cloud types	ground-based camera	support vector machine	HUST dataset	Accuracy (ACC) for different clouds: from 60% to 96%
M. P. Souza-Echer et al. [23]	Cloud existence estimation	ground-based camera	Classification of each pixel in IHS	Custom dataset	Accuracy (ACC) up to 94%
B. Nouri et al. [24]	Weather classification	ground-based camera	Convolutional neural network (CNN)	MWI and Cityscapes datasets	Accuracy (ACC) for different datasets: from 90% to 95%

**Table 2:** Parameters, used in the second stage of the algorithm for Canny edge detection.

Parameter	Value	Parameter Explanation
Lower	150	The lower boundary on the gradient values.
Upper	300	The upper boundary on the gradient values.

**Table 3:** Parameters, used for circle detection with Hough transform.

Parameter	Value	Parameter Explanation
Dp	7	The inverse ratio of the accumulator resolution to the image resolution.
MinDist	12	The minimum distance between the centers of the detected circles.
Param1	10	First method-specific parameter.
Param2	20	Second method-specific parameter.
minRadius	5	Minimum circle radius.
maxRadius	20	Maximum circle radius.

**Table 4:** Parameters, used for line detection with Hough transform.

Parameter	Value	
rho	100	Distance resolution of the accumulator in pixels
theta	π/2	Angle resolution of the accumulator in radians.
threshold	10	Accumulator threshold parameter.
minLineLength	10	Minimum line length.
maxLineGap	15	Maximum allowed the gap between points on the same line to link them.

**Table 5:** Initial dataset and its subsets.

Subset Number	Image From	Image to
1	1	6457
2	1	1420
3	1000	2500
4	2000	3500
5	3000	4500
6	4000	5500
7	4500	6457

**Table 6:** Confusion matrix, obtained by applying the proposed algorithm with initial dataset and all its subsets.

Subset Number	TP	TN	FP	FN
1	1438	4209	451	358
2	38	1341	18	22
3	33	1231	167	70
4	382	747	208	164
5	441	875	109	76
6	773	506	132	90
7	804	868	152	134

**Table 7:** Summary of classification metrics results, for different dataset subsets.

Subset Number	TPR	TNR	PPV	NPV	ACC	BA	F1-Score
1	0.801	0.903	0.761	0.922	0.875	0.852	0.781
2	0.633	0.987	0.679	0.984	0.972	0.810	0.655
3	0.320	0.881	0.165	0.946	0.842	0.601	0.218
4	0.700	0.782	0.648	0.820	0.752	0.741	0.673
5	0.853	0.889	0.802	0.920	0.877	0.871	0.827
6	0.896	0.793	0.854	0.849	0.852	0.844	0.874
7	0.857	0.851	0.841	0.866	0.854	0.854	0.849

**Table 8:** Summary of SWIMCAT-extended dataset image classification to cumulus and non-cumulus for each class.

	Clear Sky	Patterned Clouds	Thin White Clouds	Thick White Clouds	Thick Dark Clouds	Veil Clouds
Cumuliform	0	85	150	153	3	0
Non cumuliform	350	265	200	197	347	350

**Table 9:** The specifications of the UAV which will be used for the experiment.

Parameter	Value
Wingspan	4.6 m
Weight	4.3 kg
Power source	C60 Solar cells, 17,000 mAh li-ion battery
Autopilot	Pixhawk Cube Orange
Companion computer	Raspberry Pi 3 Model B
Payload	Kurakesu C1 micro camera, Gopro hero8 (for terrain mapping)
Flight Time	3–6 h (depends on weather conditions)
Operation range	5 km with standard 2.4 GHz telemetry/Unlimited with LTE telemetry

**Table 10:** The summary of simulation data and results.

Situation	Segment	Altitude at the Beginning, m	Climb Rate, m/s	Duration, s	Distance, m	Altitude at the End, m	Total Flight Time, s	Distance to the F, m
Direct flight	From S towards F	185	−0.367	231.61	3150	100	232	6850
(a)	From S to Thermal	185	−0.367	97.06	1320	149	1156	250
	Thermal	149	0.293	514.06	0	300		
	From thermal to F	300	−0.367	544.93	7411	100		
(b)	From S to Thermal	185	−0.367	231.63	3150	100	232	7070
(c)	Rotation in S	185	−0.367	2.50	0	184	1998 *	0
	From S to Thermal	184	−0.367	183.82	2500	116		
	Thermal	116	0.739	924.49	0	800		
	From thermal to F	800	−0.367	889.71	12,100	473		

* The finish was reached, while the UAV could continue the gliding and reach the 3015 s flight time, adding additional 13,830 m (enough to go back to S).

## Data Availability

The programming code is available online at https://github.com/ivansuzdalev/clouds_reco_canny_hough (accessed on 25 August 2021).
